# Defining the lipid profiles of human milk, infant formula, and animal milk: implications for infant feeding

**DOI:** 10.3389/fnut.2023.1227340

**Published:** 2023-08-30

**Authors:** Alexandra D. George, Sudip Paul, Tingting Wang, Kevin Huynh, Corey Giles, Natalie Mellett, Thy Duong, Anh Nguyen, Donna Geddes, Toby Mansell, Richard Saffery, Peter Vuillermin, Anne-Louise Ponsonby, David Burgner, Satvika Burugupalli, Peter J. Meikle, Barwon Infant Study Investigator Team, Anne-Louise Ponsonby, David Burgner, Fiona Collier, John Carlin, Katie Allen, Mimi Tang, Peter Sly, Peter Vuillermin, Richard Saffery, Sarath Ranganathan, Terry Dwyer

**Affiliations:** ^1^Metabolomics Laboratory, Baker Heart and Diabetes Institute, Melbourne, VIC, Australia; ^2^Baker Department of Cardiometabolic Health, University of Melbourne, Parkville, VIC, Australia; ^3^Department of Cardiovascular Research, Translation and Implementation, La Trobe University, Bundoora, VIC, Australia; ^4^School of Molecular Sciences, The University of Western Australia, Perth, WA, Australia; ^5^Murdoch Children’s Research Institute, Parkville, VIC, Australia; ^6^Department of Pediatrics, University of Melbourne, Parkville, VIC, Australia; ^7^School of Medicine, Deakin University, Melbourne, VIC, Australia; ^8^Child Health Research Unit, Barwon Health, Geelong, VIC, Australia; ^9^The Florey Institute of Neuroscience and Mental Health, Melbourne, VIC, Australia; ^10^Department of Diabetes, Central Clinical School, Monash University, Clayton, VIC, Australia

**Keywords:** breastfeeding, breastmilk, metabolomics, DOHaD (development origins of health and disease), fat

## Abstract

**Background:**

Breastfed infants have lower disease risk compared to formula-fed infants, however, the mechanisms behind this protection are unknown. Human milk has a complex lipidome which may have many critical roles in health and disease risk. However, human milk lipidomics is challenging, and research is still required to fully understand the lipidome and to interpret and translate findings. This study aimed to address key human milk lipidome knowledge gaps and discuss possible implications for early life health.

**Methods:**

Human milk samples from two birth cohorts, the Barwon Infant Study (*n* = 312) and University of Western Australia birth cohort (*n* = 342), were analysed using four liquid chromatography-mass spectrometry (LC–MS) methods (lipidome, triacylglycerol, total fatty acid, alkylglycerol). Bovine, goat, and soy-based infant formula, and bovine and goat milk were analysed for comparison. Composition was explored as concentrations, relative abundance, and infant lipid intake. Statistical analyses included principal component analysis, mixed effects modelling, and correlation, with false discovery rate correction, to explore human milk lipidome longitudinal trends and inter and intra-individual variation, differences between sample types, lipid intakes, and correlations between infant plasma and human milk lipids.

**Results:**

Lipidomics analysis identified 979 lipids. The human milk lipidome was distinct from that of infant formula and animal milk. Ether lipids were of particular interest, as they were significantly higher, in concentration and relative abundance, in human milk than in formula and animal milk, if present in the latter samples at all. Many ether lipids were highest in colostrum, and some changed significantly through lactation. Significant correlations were identified between human milk and infant circulating lipids (40% of which were ether lipids), and specific ether lipid intake by exclusively breastfed infants was 200-fold higher than that of an exclusively formula-fed infant.

**Conclusion:**

There are marked differences between the lipidomes of human milk, infant formula, and animal milk, with notable distinctions between ether lipids that are reflected in the infant plasma lipidome. These findings have potential implications for early life health, and may reveal why breast and formula-fed infants are not afforded the same protections. Comprehensive lipidomics studies with outcomes are required to understand the impacts on infant health and tailor translation.

## Introduction

In the first months of life, human milk provides the infant with a multitude of nutritive and bioactive components, including lipids, which make up approximately 3–5% of human milk (w/w) (1). Not only does the lipid portion provide the majority of energy (approximately 50%) to the breastfed infant, it also delivers potentially bioactive species with critical roles in early life (2–4). The human milk lipidome is complex, comprised of numerous lipid classes, including triacylglycerols, sphingolipids, gangliosides each made up of hundreds of individual lipid species, many of which can be difficult to measure (2). Of the macronutrient components, the percentage of total lipids in human milk displays the highest interindividual variation. The percent of lipids is also related to the amount of milk in the breast at the time of collection (and thus feedings patterns), resulting in high variation (5). The degree to which the specific lipid species that comprise human milk vary between individuals and over time is unclear.

Breastfed infants have better short- and long-term health outcomes than formula-fed infants (6). The compositional differences between human milk and infant formula are likely responsible, at least in part, for these effects. There is emerging evidence that the human milk lipidome contributes to some of the benefits afforded by breastfeeding, decreasing the risk of obesity, diabetes, and non-communicable diseases (7). The potential mechanisms include anti-infection and anti-inflammatory actions by fatty acids (8–11), sustenance of beige adipose tissue by alkylglycerols and thus decreased risk of obesity (12, 13), and establishment of healthy metabolism and lipid regulation by lipid metabolites such as 12,13-diHOME (14–16). Circulating lipid dysregulation is also a demonstrated key risk factor for obesity and related non-communicable diseases (17). In an analysis of over 600 plasma lipids in 1,074 infants (Barwon Infant Study), we previously found that 90% of circulating lipids at 6 months of age were significantly associated with any breastfeeding at 6 months of age (18).

Limited understanding of the human milk lipidome, and its variation, restricts the interpretation and translation of research in this field. To date, this has not been profiled in the same detail as human blood. As interest in the human milk lipidome increases, there is a critical need for improved profiling and understanding of its composition and variance. In this study, we utilised human milk samples from two birth cohorts to address these knowledge gaps, through (1) comprehensive profiling of the human milk lipidome, (2) comparison of human milk with infant formula and animal milk, (3) assessment of inter-and intra-individual variation, (4) investigation of concentration, relative abundance, and intake, and (5) assessment of human milk and corresponding infant circulating lipids at 6 months. We discuss the potential implications of our findings, and the future direction of human milk lipidomics to further enhance understanding, interpretation, and translation of lipidomics in this field.

## Methods

### Cohort samples

The Barwon Infant Study (BIS) is a birth cohort study assembled using an unselected antenatal sampling frame in the Barwon region of Victoria, Australia (19). Women were recruited during pregnancy and excluded from the study if infants were born premature or developed serious illness. The human milk for BIS included samples collected at 1 month (*n* = 247), 6 months (*n* = 32), and 12 months (*n* = 33), from women who were breastfeeding (exclusively or mixed, a total of 287 dyads). Pre-feed samples were collected from one breast, at the start of each visit or at the end (approximately 2 hours) if the infant was recently fed. Participants were given the option to manually express or to use a breast pump for collection of samples. Lipidomics profiling has been reported previously on infant plasma at ages 6 and 12 months (18). Ethics approval was obtained by the Barwon Health Human Research and Ethics Committee (HEC 10/24).

The University of Western Australia Longitudinal Cohort (UWAC) is a birth cohort from Perth, Western Australia, Australia. Women who intended to exclusively breastfeed for 6 months were recruited for this study during pregnancy and excluded if infants developed serious illness or were no longer exclusively breastfeeding at 6 months (20). The UWAC included 17 healthy exclusively breastfeeding mother-infant dyads. Monthly sample and growth data was collected at birth (*n* = 16), 1 month (*n* = 54), 2 months (*n* = 50), 3 months (*n* = 104), 4 months (*n* = 33), 5 months (*n* = 40), and 6 months (*n* = 45). Monthly sample types include morning, noon, and evening samples, and pre- (*n* = 60) and post-feed (*n* = 44) samples at 3 months post-partum, providing coverage of known sources of lipid variation. In month three, daily infant milk intake was also measured with 24 h test weighing (21). Ethics approval was obtained by The UWA Human Research Ethics Office (RA/4/20/4023), and all participants provided informed written consent. All human milk samples were stored refrigerated (4°C, BIS) or frozen (<0°C, UWAC) for <24 h before being transferred to a laboratory freezer (−80°C) for storage until thawing (at room temperature) and preparation for analysis.

### Other samples for comparison

Commercially available infant formula was included for comparison with human milk, bovine milk-based (*n* = 6), goat milk-based (*n* = 2) and soy based (*n* = 2). Each formula type was reconstituted in water as per the directions. The fat from each of these infant formula samples is derived predominantly from vegetable and plant oils. Commercially available bovine (*n* = 2) and goat milk (*n* = 1) were also included for comparison.

### Lipid extraction

Single phase lipid extraction is commonly carried out using a chloroform methanol method, however, to reduce preparation time and increase throughput (amenable to automation), we used single phase butanol and methanol extraction, after establishing efficacy (Supplementary File S1 and Table S1) (22). Lipids were extracted from 10 μL samples using 100 μL extraction solvent butanol: methanol (1,1, v/v) containing 10 mM ammonium formate and internal standards. Samples were vortexed, sonicated for 1 h, centrifuged for 10 min (14,000x g, 20°C), and supernatant was transferred to 2 mL glass mass spectrometry vials with 250 μL inserts (Agilent Technologies) for analysis. All sample types were extracted using butanol: methanol, with some alterations (described for each analysis method in Supplementary Files S2–S5).

### Liquid chromatography-mass spectrometry based lipidomics

Human milk, infant formula, and animal milk samples were analysed using a combination of four liquid chromatography-mass spectrometry based lipidomics methods, as per Table 1. All methods were targeted, using scheduled multiple reaction monitoring (MRM), as described below. For all methods, analyses were performed as single batches, with quality control samples (pooled plasma QC, pooled human milk QC, and blanks) included every 20 samples. Species were identified based on MRM precursor/product ion pairs and retention time, and chromatographic peaks were integrated manually using Mass Hunter (B.09.00, Agilent Technologies) software. The median blank concentrations were subtracted from each sample. Concentrations below the limit of detection were replaced by half the minimum measured value for that species. For subsequent statistical analyses, results from method 1 and 2 were combined (representing the whole lipidome), while results from methods 3 and 4 were both kept separate.

**Table 1:** Description of the four LC–MS methods used to achieve comprehensive analysis of samples.

Method	Description	Samples analysed
Lipidome	Total lipidome excluding triacylglycerols	BIS human milk, UWAC human milk, animal milk, infant formula
Triacylglycerol	Extended method to cover triacylglycerols	BIS human milk, UWAC human milk, animal milk, infant formula
Total fatty acid	Total fatty acids that comprise the lipidome	BIS human milk, animal milk, infant formula
Alkylglycerol	Alkylglycerol composition, from alkyldiacylglycerols (TG(O))	BIS human milk, animal milk, infant formula

#### Lipidome LC–MS analysis

Lipidome analysis was carried out on an Agilent 1290 UHPLC system coupled with an Agilent 6495C triple quadrupole mass spectrometer (Supplementary File S2). Samples were extracted with butanol:methanol, as in *Lipid extraction*. Concentrations for each lipid species were calculated based on area under the chromatographic curve relative to the labeled internal standard concentrations (23). For the UWAC samples, the chromatography was extended and retention time windows shifted appropriately, to include lower-abundance short chain fatty acid containing TG (SCFA-TG) with the lipidome.

#### Triacylglycerol LC–MS analysis

Because the concentration of TG in milk were high relative to other lipid species, a separate analysis (Supplementary File S3) for TG was performed whereby samples were diluted (1 in 100) with milliQ water before lipids were extracted from 10 μL with butanol:methanol, as described above. Analysis of milk triacylglycerols was performed on an Agilent 6490 QQQ mass spectrometer with an Agilent 1290 series UHPLC system. Concentrations of each triacylglycerol were calculated based on chromatographic peak area relative to the labelled triacylglycerol internal standard (23).

#### Total fatty acid LC-MS analysis

For the analysis of total fatty acids, milk samples were saponified to release all fatty acids prior to analysis (Supplementary File S4). Mass spectrometry analysis was as described for method 1. Concentrations of each fatty acid was calculated based on chromatographic peak area relative to deuterated fatty acid internal standard concentrations.

#### Alkylglycerol LC–MS analysis

Because we noted that the amount of TG(O) species was significant in human milk, we also analysed the alkylglycerol composition to quantitate the total TG(O) species. Lipid extracts for alkylglycerol analysis were saponified (Supplementary File S5), generating alkylglycerols from ether lipids, predominantly TG(O). Mass spectrometry analysis was as described for method 1. For quantification of alkyl glycerol species, a deuterated monoacylglycerol (MG 18:1d7) was used as an internal standard. Response factors for alkylglycerol species against MG 18:1d7 were calculated using serially diluted synthetic alkyl glycerol species in a range 1–300 μM and a fixed amount of MG 18:1d7. The efficiency of saponification was assessed by the residual triacylglycerol in saponified samples.

### Infant intake comparison

Due to the complications introduced by sampling (and thus high variability between samples), infant lipid consumption (intake) was assessed at 3 months, comparing exclusively breastfed infants (UWAC) and an exclusively formula-fed infant. For the exclusively breastfed infants, total intake (in mL) was multiplied by mean sample concentration (pmol/mL) at 3 months (24). For an exclusively formula-fed infant, sample concentrations (pmol/mL) were factorised by the number and volume of feeds (at 3 months) on bovine milk infant formula package. Lipid intake was expressed as pmol/day.

### Statistical analyses

Due to the complexity of human milk lipids, lipidomic measurements were expressed as concentrations, relative abundance (proportion of the total lipid content, calculated from the molar concentrations), and intake (per day, at 3 months post-partum). Paired *t*-tests were used to compare lipid extraction methods (butanol:methanol with chloroform:methanol). Full lipidome and extended triacylglycerol results were combined for analyses, but fatty acid and alkylglycerol analyses were performed independently. Concentration and relative abundance values were log transformed prior to modelling. Principal component analysis was performed on lipidomic measures for all sample types, to visualise the major axes of variation. Unpaired *t*-tests were used to compare mean ether lipid content in the different sample types and assess if they were different. Linear mixed-effects models were used to identify trends in human milk lipids between time points (time of day, pre- or post-feed, and month post-partum). Human milk lipid (class or species) concentration or relative abundance were modelled, with sample timing as a fixed effect and individual ID was a random effect to account for intra-individual variation. Pearson correlation was performed on 637 matched BIS plasma lipids measured at 6 months of age (previously published (18)), and BIS human milk lipids measured at 1 month. Pearson correlation was also performed between the DHA-containing TG and linoleic acid (LA)-containing TG ratio in human milk and infant plasma. Linear regression was performed to compare the intakes of exclusively breastfed and exclusively formula-fed infants at 3 months of age, with results expressed as fold-differences. Benjamini and Hochberg adjustment was made to account for multiple comparisons in all analyses (false discovery rate, FDR), with adjusted *p* < 5 × 10^−2^ considered significant (25). Statistical analyses were conducted using R Studio (version 4.1.2). Unless otherwise stated, values are presented in the text as mean ± standard deviation (SD).

## Results

A total of 312 milk samples were analysed from BIS (Supplementary Table S2) with each of the four methods (Table 1). At the 1 month milk sample collection, 34.2% (67/196 who answered) reported exclusively breastfeeding, at 6 month collection 6.7% (2/30 who answered) reported exclusively breastfeeding, and at 12 months all participants (excluding one) were breastfeeding at least one feed per day. UWAC (Supplementary Table S3) was comprised of exclusively breastfeeding dyads and was used to look comprehensively at sampling and longitudinal human milk trends. A total of 342 longitudinal samples from UWAC were analysed.

### Liquid chromatography-mass spectrometry allows comprehensive human milk lipidomics

Combining the lipidome and triacylglycerol LC–MS methods (methods 1 and 2), allowed measurement of 979 lipid species (Supplementary Table S4). The median human milk QC CV for each method was 8.8 and 11.7%, respectively, and all CV were <20%. In all human milk samples, approximately 99% of the total lipidome (w/w) was comprised of triacylglycerols (74 ± 17% of the total lipidome), free fatty acids (12 ± 10%), diacylglycerols (11 ± 6%), and monoacylglycerols (2 ± 1%).

Samples were saponified and total fatty acids were measured (method 3), from C8:0 to C24:6 (Supplementary Table S5). The median CV for the fatty acid method was 14.5%, and all CV were <20%. The total fatty acid component of human milk was comprised primarily of C18:1 (40 ± 5%), with C16:0 (18 ± 2%), C18:2 (8 ± 2%), and C18:0 (8 ± 2%), C14:0 (7 ± 3%), C12:0 (6 ± 2%), and all others comprising ≤2% of the total fatty acids measured.

Similarly, alkylglycerols (method 4) (from TG(O)) were also measured, via sample saponification (Supplementary Table S6). The median CV for the alkylglycerol method was 15.4%, and all CV were <26%. This method was able to identify 18 alkylglycerol (AKG) species, ranging from AKG(12:0) to AKG(24:1). The three most abundant species (AKG(18:1), AKG(16:0), and AKG(18:0)) comprised over 90% of the total AKG content.

### Human milk has a distinctive lipidome

**Figure 1:**
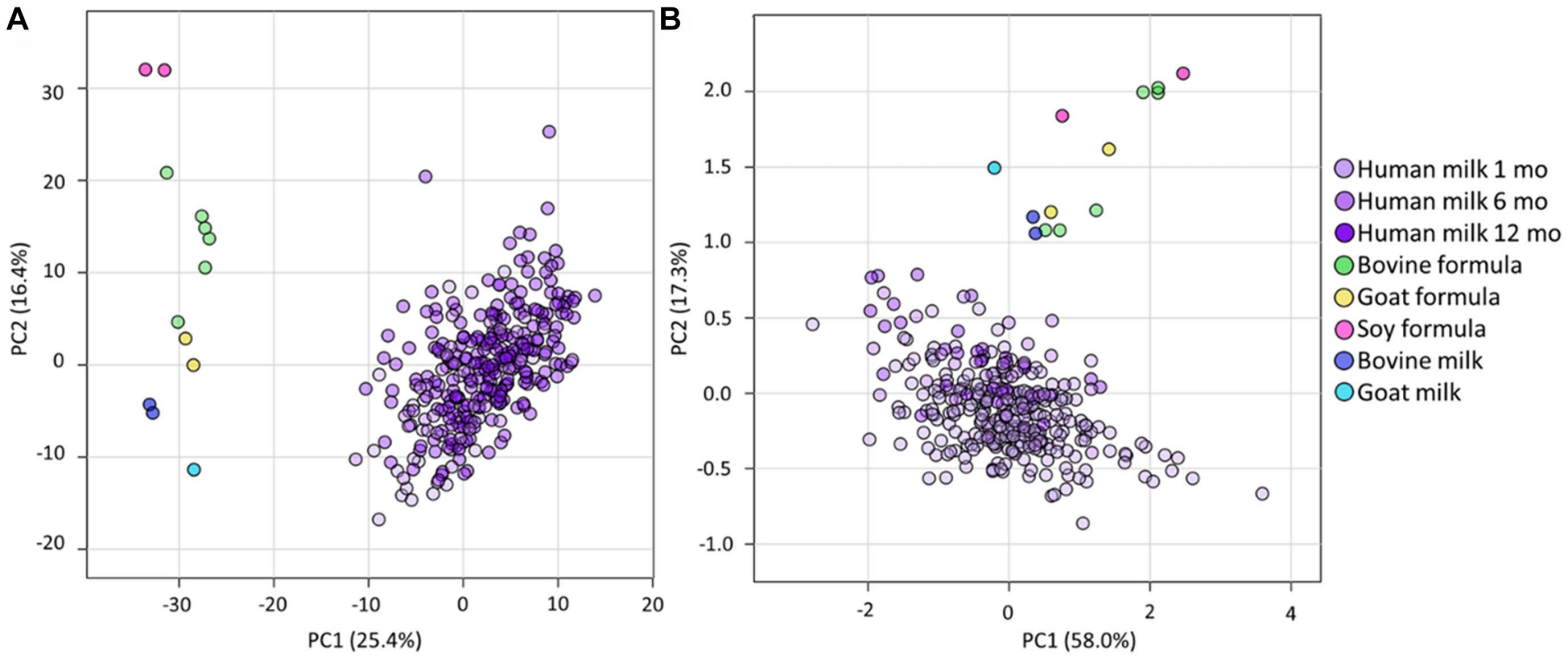
The lipidomes of human milk (BIS), infant formula, and animal milk. PCA of lipidomic measures for **(A)** the total lipidome and **(B)** the total fatty acid composition, for BIS human milk at 1 (Human milk 1 mo), 6 (Human milk 6 mo), and 12 (Human milk 12 mo) months, infant formula (bovine, goat, and soy), and animal milk samples (bovine milk and goat milk). All concentrations were log10 transformed prior to PCA.

Principal component analyses (PCA) was firstly used to obtain an overview of the relatedness of the lipid profiles of the infant formula and animal samples analysed in this study. This showed clear separation of all the human milk samples from all infant formula, and animal milk samples, for both total lipid species (Figure 1A) and total fatty acids (Figure 1B). The lipidome of infant formula of bovine, goat, and soy milk origin, and bovine and goat milk, differed from each other (Figure 1A), but clustered closer together when total fatty acid composition was assessed (Figure 1B).

**Figure 2:**
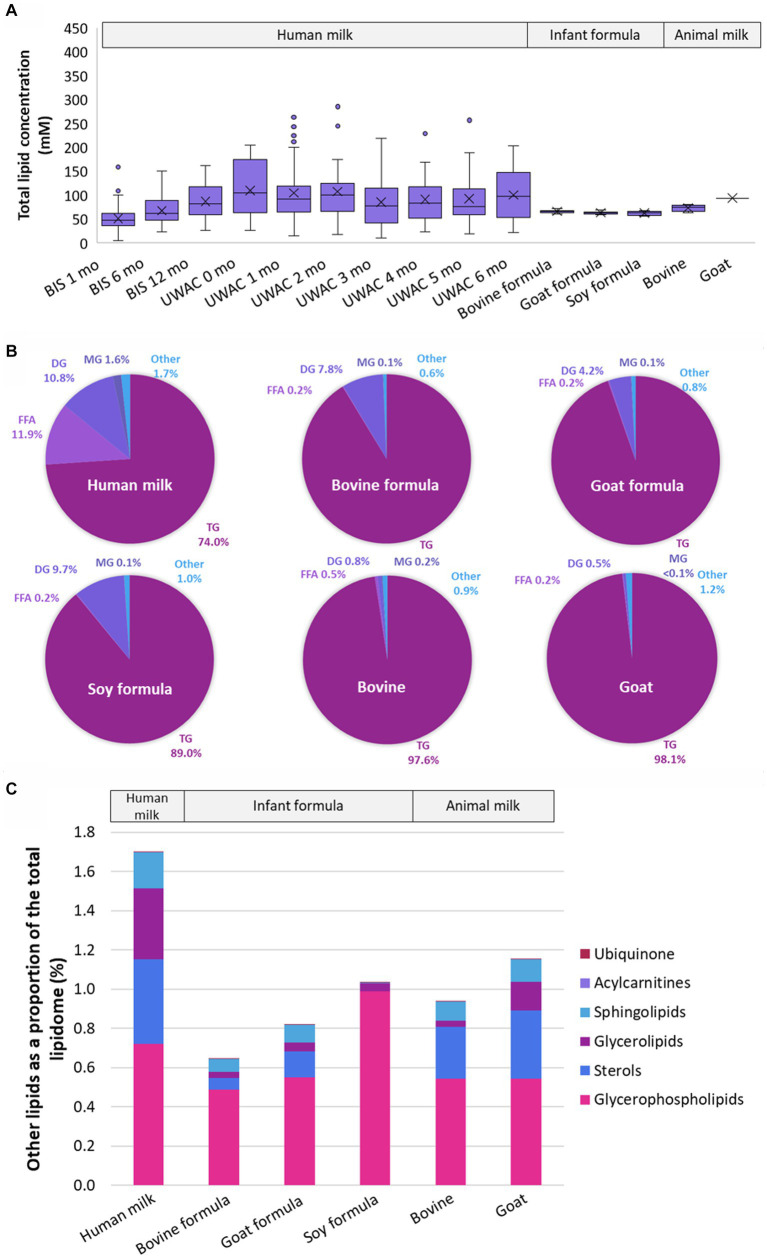
The lipidomes of human milk (*n* = 654), bovine (*n* = 6), goat (*n* = 2), and soy (*n* = 2) based infant formula (*n* = 6), bovine milk (*n* = 1), and goat milk (*n* = 1). **(A)** The total lipid concentration (mM) of animal milk, infant formula, and human milk samples. Boxes are median and lower (25%) and upper (75%) quartile values, crosses are mean values, whiskers are minimum and maximum, with outliers greater than 1.5× the interquartile range. **(B)** The mean relative abundance of lipids that comprise the human milk, bovine milk, goat milk, bovine milk formula, goat milk formula, and soy milk formula lipidomes, represented as class totals triacylglycerol (TG), free fatty acid (FFA), diacylglycerol (DG), monoacylglycerol (MG) and Other. **(C)** The mean relative abundance (as a percentage of the total lipid content, calculated from molar concentrations) of other lipid classes in human milk, bovine milk, goat milk, bovine milk formula, goat milk formula, and soy milk formula. Lipid classes are listed in ascending order of magnitude for human milk and defined as Ubiquinone, Acylcarnitines (hydroxylated acylcarnitine and acylcarnitine), Sphingolipids (sphingomyelin, monohexosylceramide, dihexosylceramide, trihexosylceramide, ceramide, deoxyceramide, dihydroceramide, GM3 ganglioside, sphingosine, and sulfatide), Glycerolipids (alkyldiacylglycerol and monoalkyldiacylglycerol), Sterols (free cholesterol and cholesterol ester), Glycerophospholipids (phosphatidylcholine, phosphatidylinositol, phosphatidylethanolamine, alkenylphosphatidylethanolamine, phosphatidylserine, lysophosphatidylethanolamine, lysophosphatidylcholine, alkylphosphatidylcholine, alkenylphosphatidylcholine, lysophosphatidylinositol, phosphatidic acid, alkylphosphatidylethanolamine, lysoalkenylphosphatidylethanolamine, phosphatidylglycerol, lysoalkylphosphatidylcholine, and lysoalkenylphosphatidylcholine). Ubiquinone and Acylcarnitines comprise <0.002% in all sample types. Triacylglycerol, diacylglycerol, monoacylglycerol, and free fatty acid classes were not included.

Despite the total lipid content being very similar in all sample types (all *p* > 5 × 10^−2^, Figure 2A), there were marked compositional differences between human milk, infant formula, and animal milk, at both the lipid species and class level (Figures 2B,C and Supplementary Table S4). Human milk contained lower TG than other samples, however the sum of TG, FFA, DG, MG was 98.3% of the total lipid content, approximately 0.6–1.1% lower than bovine formula (99.4%), goat formula (99.2%), soy formula (99.0%), bovine milk (99.1%), and goat milk (98.9%). The ‘other’ lipid classes (Figure 2C) in infant formula and animal milk were particularly different from human milk. Specifically, the three most abundant ‘other’ classes in human milk comprised >0.9%, made up of COH (0.4%), TG(O) (0.3%), and PC (0.2%). In contrast, these three classes comprised 0.3% of bovine formula, 0.4% of goat formula, and 0.5% soy based formula, with PC making up the majority of this (0.2, 0.3, and 0.5% respectively). Measures for the animal milk samples were closer to that of human milk, with COH, TG(O), and PC comprising 0.5% of bovine milk and 0.7% of goat milk, with majority comprising COH (0.3%).

Large differences in both concentration and relative abundance were also reflected in the FA analysis. The total fatty acid content varied significantly from human milk (106.3 ± 45.5 mM) and bovine formula (135.4 ± 0.1 mM, *p* = 1.4 × 10^−2^), soy formula (169.2 ± 76.3 mM, *p* = 4.2 × 10^−3^), and goat milk (166.6 mM, *p* = 2.3 × 10^−2^), but not from goat formula (135.4 ± 10.1 mM, *p* = 1.2 × 10^−1^), or bovine milk (117.5 ± 5.5, *p* = 0.5.5 × 10^−1^). C18:1 was the most prevalent fatty acid in all sample types, comprising 40% of the total fatty acid content of human milk, 37% of bovine and soy milk formula, and 45% of goat milk formula. C18:1 made up 24 and 26% of goat and bovine milk fatty acids, respectively (Supplementary Table S5). Odd-chain fatty acids, including C15:0, C17:0, and C19:1, were all lower in concentration in infant formula, regardless of milk base.

### Human milk contains high levels of ether lipids

**Figure 3:**
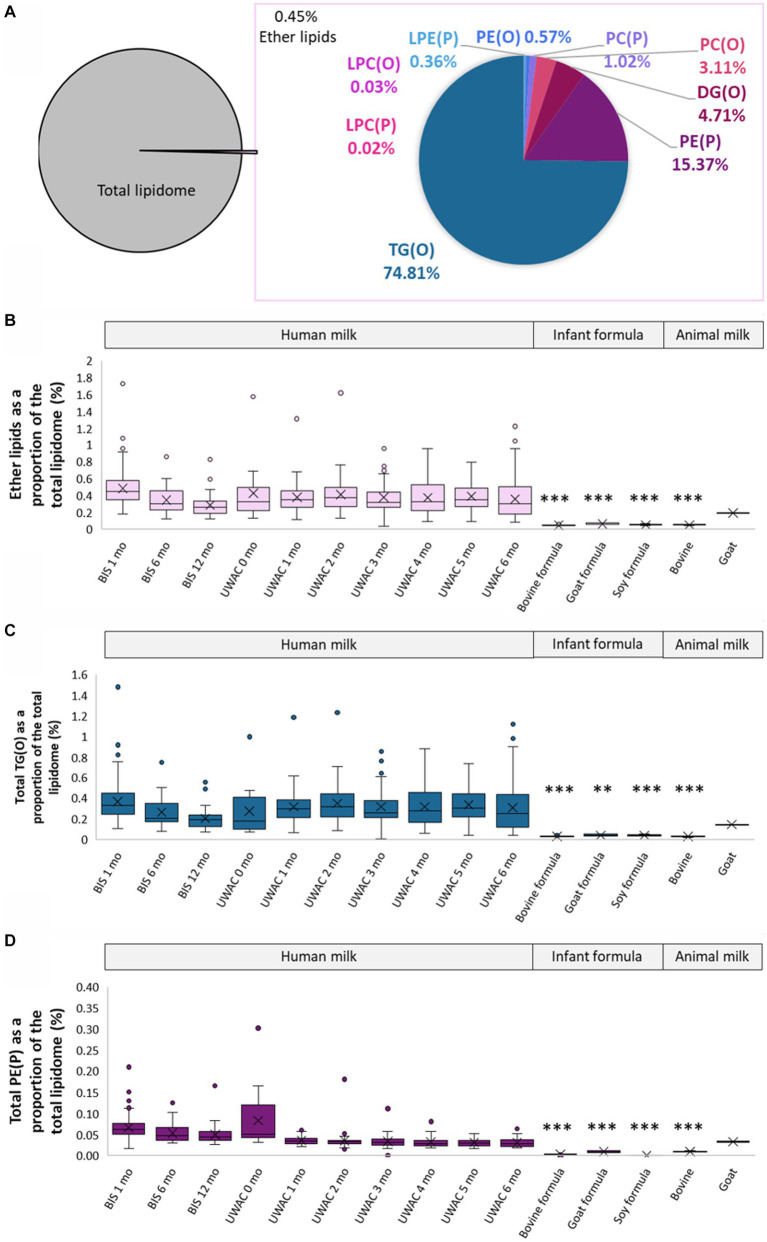
Ether lipids are different in human milk, infant formula, and animal milks. **(A)** Total ether lipids as a proportion of the total lipidome, and the proportion of each class of ether lipids. **(B)** Total ether lipids as a proportion of total lipidome (%, calculated using the molar concentrations) in BIS and UWAC human milk samples, infant formula, and animal milk. **(C)** Total TG(O) as a proportion of total lipidome (%) in BIS and UWAC human milk samples, infant formula, and animal milk. **(D)** Total PE(P) as a proportion of total lipidome (%) in BIS and UWAC human milk samples, infant formula, and animal milk. Boxes are median and lower (25%) and upper (75%) quartile values, crosses are mean values, whiskers are minimum and maximum, with outliers greater than 1.5× the interquartile range. Significant differences between the mean values of human milk and other sample types are represented by *(*p* < 0.05), **(*p* < 0.01), and ***(*p* < 0.001).

Ether lipids, those containing an ether or vinyl ether link, made up approximately 0.45 ± 0.19% of the total human milk lipidome, comprised of TG(O) (0.28 ± 0.09%), PE(P) (0.06 ± 0.03%), DG(O) (0.02 ± 0.02%), PC(O) (0.01 ± 0.003%), PC(P) (0.004 ± 0.002%), LPE(P) (0.001 ± 0.002%), LPC(O) (0.0001 ± 0.0001%), and LPC(P) (0.00009 ± 0.00007%) (Figure 3A). In contrast, total ether lipids comprised approximately 0.05 ± 0.005% bovine milk, 0.19% goat milk, 0.04 ± 0.004% bovine milk-based infant formula, 0.06 ± 0.009% goat milk-based infant formula, and 0.05 ± 0.007% soy-based infant formula (Figure 3B).

TG(O) comprised the majority of the ether lipids in human milk and were found in significantly higher proportions than in infant formula and bovine milk (all *p* < 0.0001), but were not different in goat milk (*p* > 0.05) (Figure 3C). Over 80% of the TG(O) class was made up of 10 species, including TG(O-52:2), TG(O-52:1), TG(O-50:1), TG(O-54:3), and TG(O-54:2), containing primarily C18:1 and C16:0 fatty acids. PE(P) lipids (Figure 3D) were the second most abundant ether lipid, comprising 15% of total ether lipid content. Sixteen different PE(P) species made up over 80% of the total PE(P), containing many polyunsaturated fatty acid species (PUFA) (Supplementary Table S4).

**Figure 4:**
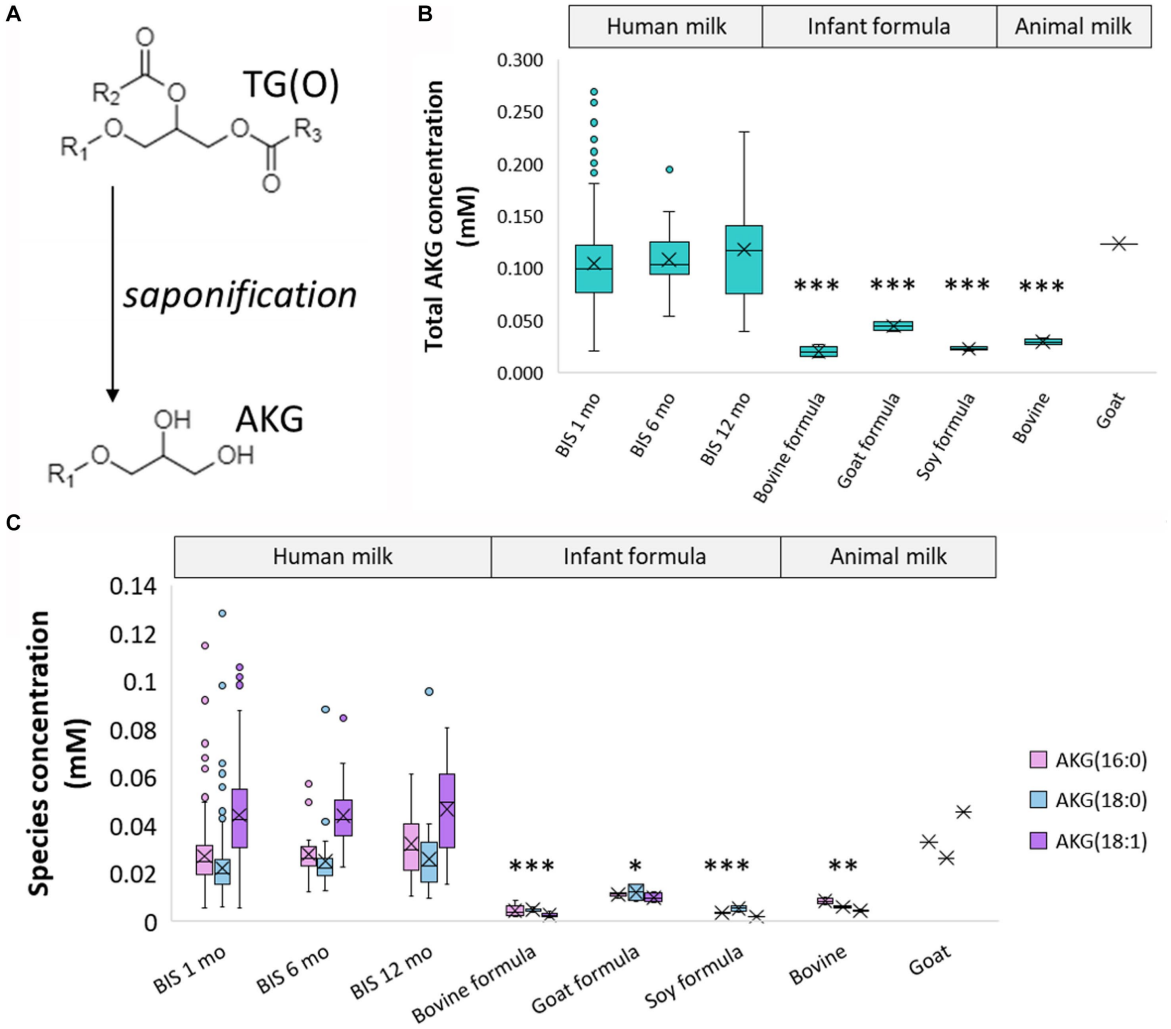
Alkylglycerol analysis of human milk TG(O). **(A)** Akyldiacylglycerol (TG(O)) were saponified to alkylglycerol (AKG) for further measurement. **(B)** Total concentration of the alkylglycerol content of human milk, infant formula, and animal milk. Significant differences between human milk and other sample types are represented by *(*p* < 0.05), **(*p* < 0.01), and ***(*p* < 0.001). **(C)** Concentration of the three most abundant AKG in human milk, infant formula, and animal milk. The lowest significant differences between each AKG in human milk and other sample types are represented by *(*p* < 0.05), **(*p* < 0.01), and ***(*p* < 0.001). Boxes are median and lower (25%) and upper (75%) quartile values, crosses are mean values, whiskers are minimum and maximum, and single points are outliers greater than 1.5× upper quartile.

To have a more accurate measurement of the TG(O) species, milk samples were saponified to measure the alkylglycerol content (Figure 4A). These alkylglycerols originate from TG(O) species, and were made up of predominantly AKG(18:1), AKG(18:0), and AKG(16:0) (Figure 4C) (Supplementary Table S6). Compared to human milk 0.12 ± 0.1%, all infant formula (0.001–0.005%), bovine milk (0.004%), and goat milk (0.007%) all contained lower levels of AKG. However, goat milk contained concentrations closest to human milk (0.12 mM versus 0.11 ± 0.04 mM respectively) (Figure 4B).

### The lipidome of human milk differs over lactation

#### Human colostrum has a distinct lipidome

Results for colostrum samples (UWAC) were analysed separately and compared to 1 month human milk samples. Total lipid content was similar in colostrum and mature milk (colostrum: 108 ± 59 mM vs. 1 month: 105 ± 61 mM; *p* > 5 × 10^−2^), yet there were distinct lipid class and species differences (Supplementary Tables S7, S8). For reference, 50–100 mM total human milk lipids is equivalent to 3–7 g/100 mL. Specifically, at the class level, colostrum was higher in TG(O) (1.3 times), LPE(P) (5 times), LPC(P) (3.3 times), PE(P) (2 times), and PC (2.5 times) (all *p* < 5 × 10^−2^). Only GQ1 (4.23-fold), FFA (3.20-fold), BA (2.75-fold), and DG (2.62-fold) increased significantly from colostrum to mature milk (all *p* < 5 × 10^−2^). The concentration of 35% of the individual species were significantly different between colostrum and one-month mature milk, which included many non-TG(O) ether lipids.

#### The human milk lipidome varies in concentration but not composition throughout a feed

There were significant differences between lipid species concentrations pre-feed and post-feed at 3 months post-partum (Supplementary Tables S9, S10). The concentration of 46% of the lipid species increased significantly (FDR adjusted *p* < 5 × 10^−2^), and one (TG(O-54:4)) decreased significantly (*p* = 3.74 × 10^−2^). Similarly, 11 out of 49 lipid class totals increased significantly (*p* < 5 × 10^−2^). For this reason, only pre-feed samples were included in the subsequent analyses. However, when we compared relative abundance, only 9% of the lipid species changed significantly, and no significant class changes were observed between pre- and post-feed samples (Supplementary Tables S11, S12).

#### The human milk lipidome does not change throughout the day

While 18 of the lipid classes increased significantly in concentration throughout the day, only 4 species (dhCer(d18:0/24:0), PC(P^−18^:1/22:6), AC(18:1), and PC(O-16:0/22:6)) did (all *p* < 5 × 10^-2^, Supplementary Tables S13, S14). When relative abundance was modelled, no significant changes occurred for the lipid classes, and only 3 species (dhCer(d18:0/24:0), PC(O-42:5), and PC(O-44:6)) increased (all *p* < 5 × 10^−2^, Supplementary Tables S15, S16).

#### The human milk lipidome changes significantly throughout lactation

**Figure 5:**
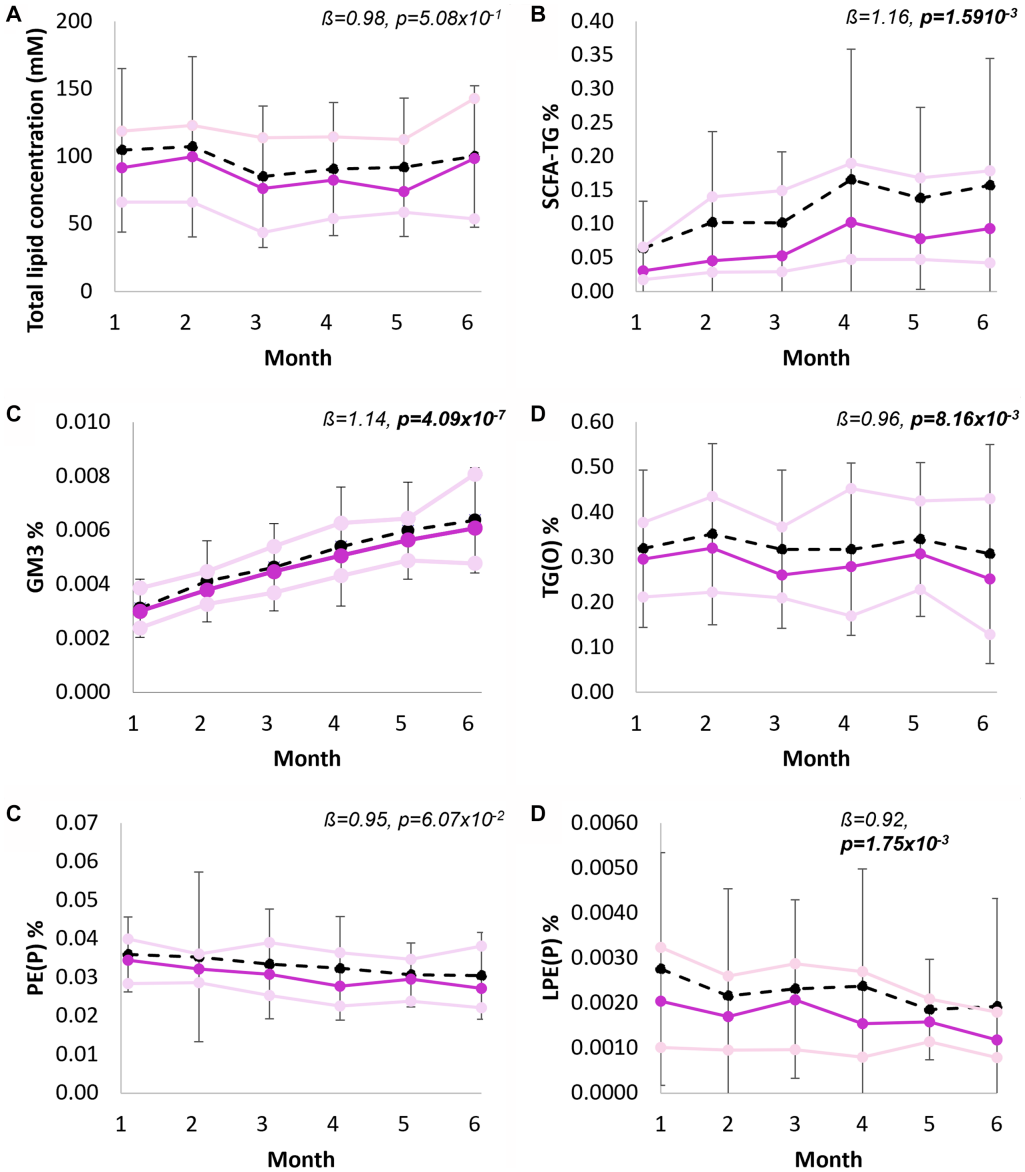
Longitudinal trends of human milk lipid classes, from 1 to 6 months exclusive breastfeeding. **(A)** Total lipid concentration of human milk (mM), **(B)** Short chain fatty acid containing TG as percentage of total lipid content, **(C)** Total GM3 gangliosides as percentage of total lipid content, **(D)** Total TG(O) as percentage of total lipid content, **(D)** Total PE(P) as percentage of total lipid content, and **(E)** Total LPE(P) as percentage of total lipid content. Black line indicates mean and standard deviation, purple line indicates median, pink lines indicate first and third quartiles. Interpretation of the beta coefficient is fold change per month, *p* values are FDR corrected (*p* < 0.05 in bold). Percentage of total lipid content was calculated from molar concentrations.

Both absolute concentration and relative abundance of human milk lipids were compared across 6 months lactation. The total lipid content did not change over the full lactation period (*p* = 5.08 × 10^−1^; Figure 5A). However, significant concentration changes occurred for sixteen of the lipid classes, eight classes increased significantly from 1 to 6 months, and eight classes decreased significantly from months one to six (Supplementary Table S17). The greatest concentration increases per month were measured for GQ1 (1.24-fold, *p* = 2.33 × 10^−21^), dimethyl-CE (1.19-fold, *p* = 1.86 × 10^−3^), SCFA-TG (1.16-fold, *p* = 1.59 × 10^−3^), and GM3 (1.14-fold, *p* = 4.09 × 10^−7^), while ether lipids including LPE(P) (*p* = 1.75 × 10^−3^), LPC(O) (*p* = 6.47 × 10^−3^), and TG(O) (*p* = 8.16 × 10^−3^), were all lower each month (all approximately 1.1 times). Other ether lipids, such as PE(P), did not change significantly through lactation (0.95-fold, *p* = 6.06 × 10^−2^). At the species level, 171 lipids changed significantly, with most, 121/171, decreasing (Supplementary Table S18). The analysis of lipid classes as a proportion of the total lipid content (Supplementary Table S19) revealed similar findings, increases for SCFA-TG (from approximately 0.06 to 0.16% of the total lipid content, Figure 5B), GM3 (increased from 0.003 to 0.006%, Figure 5C), no change to TG(O) (Figure 5D), and decreases to PE(P) (0.035 to 0.027%, Figure 5D) and LPE(P) (0.00219 to 0.00143%, Figure 5E). At the species level (Supplementary Table S20), 180 species changed significantly, approximately half decreasing (93/180) and half increasing (87/180).

#### Human milk lipids correlate with plasma lipids

Matched lipids were compared between the BIS human milk samples at 1 month lactation and corresponding infant plasma samples at 6 months of age. Significant positive correlations (*p* < 5 × 10^−2^) existed between the relative abundance (as a proportion of the total lipid content) of 122 of 637 lipid species (uncorrected), and 51 of 637 after FDR correction. This included primarily lipid species containing PUFAs, such as 22:6, 22:5, and 20:5. Notably, 40% of the significantly correlated lipids in human milk and plasma, after FDR correction, were ether lipids (Supplementary Table S21). The ratio of DHA-containing TG to linoleic acid (LA)-containing TG was positively correlated between milk and infant plasma (Pearson correlation = 0.37, *p* = 3.43 × 10^−7^).

#### Exclusively breastfed infants have a different lipid diet to that of exclusively formula-fed infants

**Figure 6:**
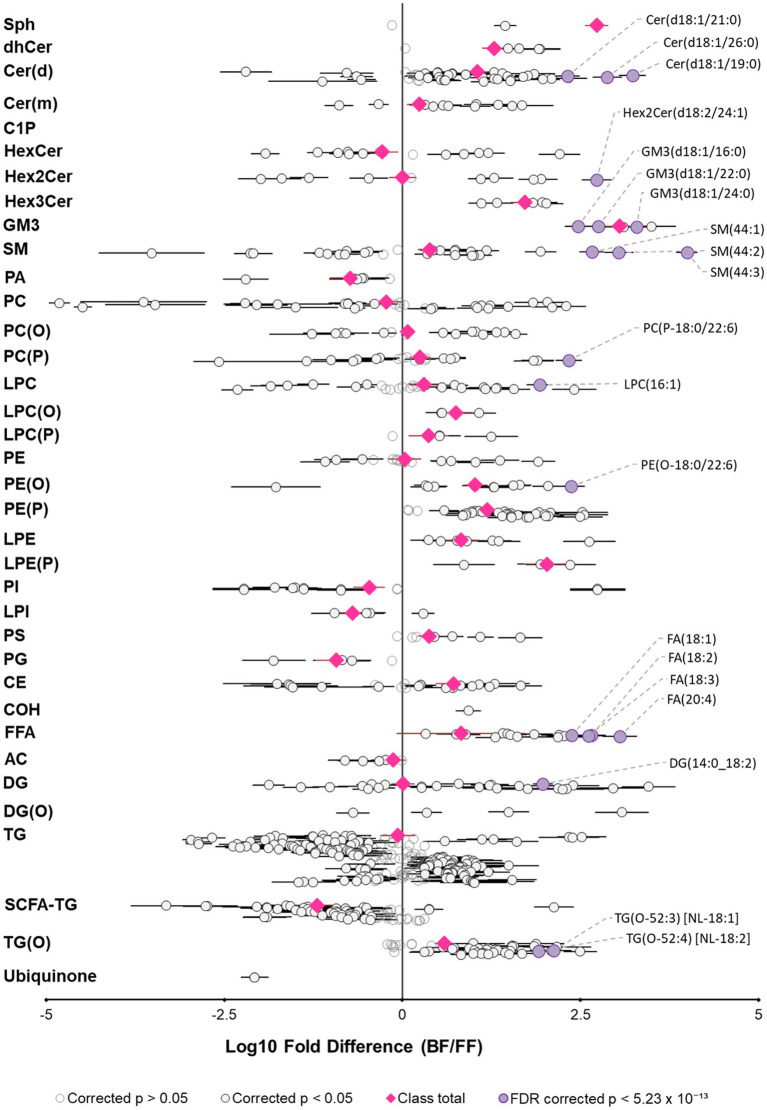
Exclusively breastfed and formula-fed infants consume different lipid diets. Fold difference of lipid intake (matched lipids) for exclusively breastfed infants relative to an exclusively formula-fed infant at 3 months of age. Each circle represents an individual lipid species, grey open circles represent *p* > 0.05, white closed circles represent *p* < 0.05, the top 20 lipid species that are higher for breastfed infants are shown in purple and labeled. Pink circles represent lipid class totals. All *p* values were corrected for multiple comparisons (Benjamini and Hochberg adjustment). Horizontal bars indicate 95% confidence intervals for significant species. Note, plotted species exclude lipids that were not measured in both sample types. Lipid classes are listed in biological order, abbreviations are sphingosine (Sph), dihydroceramide (dhCer), ceramide (Cer(d)), deoxyceramide (Cer(m)), ceramide-1-phosphate (C1P), monohexosylceramide (HexCer), dihexosylceramide (Hex2Cer), trihexosylceramide (Hex3Cer), ganglioside GM3 (GM3), sphingomyelin (SM), phosphatidic acid (PA), phosphatidylcholine (PC), alkylphosphatidylcholine (PC(O)), alkenylphosphatidylcholine (PC(P)), lysophosphatidylcholine (LPC), lysoalkylphosphatidylcholine (LPC(O)), lysoalkenylphosphatidylcholine (LPC(P)), phosphatidylethanolamine (PE), alkylphosphatidylethanolamine (PE(O)), alkenylphosphatidylethanolamine (PE(P)), lysophosphatidylethanolamine (LPE), lysoalkenylphosphatidylethanolamine (LPE(P)), phosphatidylinositol (PI), lysophosphatidylinositol (LPI), phosphatidylserine (PS), phosphatidylglycerol (PG), cholesterol ester (CE), free cholesterol (COH), free fatty acid (FFA), acylcarnitine (AC), diacylglycerol (DG), monoalkyldiacylglycerol (DG(O)), triacylglycerol (TG), SCFA-containing triacylglycerol (SCFA-TG), alkyldiacylglycerol (TG(O)).

To further understand infant lipid dietary differences between human milk and infant formula, in the context of early life, we compared the infant lipid intake at 3 months, for an infant exclusively breastfed or exclusively formula-fed. This was calculated with concentrations and the milk intake from UWAC, and infant formula preparation instructions. Firstly, milk intakes for exclusively breastfeeding infants were 741 ± 163 mL/day, while formula intake was 850 ± 77 mL/day. Overall specific lipid species intake varied widely between breastfed infants and separated distinctly from that of exclusively formula-fed infants, however total lipid intake was not different (*p* = 9.2 × 10^−1^). Significant differences existed between most lipid classes (Figure 6, Supplementary Tables S22, S23), excluding only total PC, FFA, AC, PC(O), TG, PE, DG, and Hex2Cer. Most lipid classes were consumed in higher amounts by exclusively breastfed infants. Almost all ether lipid species were fed to the exclusively breastfed infant in significantly higher amounts, however, this analysis excluded those species that were not able to be measured in infant formula.

## Discussion

It is critical that human milk lipidomics continues to improve and advance, in order to effect meaningful research interpretation and translation, to understand and improve early life health. In this study we profiled the lipidome of 654 human milk samples from two birth cohorts, BIS and UWAC, to advance current lipidome understanding. The key findings from this study were (1) the human milk lipidome differs from that of infant formula, animal milk, and is rich in ether lipids, (2) human milk lipids exhibit longitudinal trends, and (3) the human milk lipidome impacts infant circulating lipids.

### The human milk lipidome differs from that of infant formula, and animal milk, and is rich in ether lipids

Marked lipidome differences were identified between human milk samples and infant formula and animal milk, with distinct separation for both the lipidome (Figure 1A) and the total fatty acid composition (Figure 1B). This was despite the total lipid concentration being similar for human milk and infant formula samples (Figure 2A), which is likely a reflection of energy requirements for formulation of infant food. The clear difference between animal milk and corresponding infant formula is also likely a reflection of preparation processes for infant formula, removing a large portion of the native lipids and/or adding a blend of vegetable oils. This human milk analysis adds many additional species to existing works, including 204 ether lipids from PC(O), PC(P), LPC(O), LPC(P), PE(O), PE(P), DG(O), and TG(O) classes, which are low abundance and thus difficult to measure (2, 10, 20, 26–29). At the lipid class level (Figure 2B), TG comprise the majority of human milk and all infant formula, although the higher proportion of FFA, DG, and MG in human milk may be a result of triacylglycerol lipolysis in human samples (infant formula was made fresh for analysis and does not contain lipase enzymes). Nevertheless, the ‘other’ portion of the lipidome comprises approximately 1.7% of human milk, and between 0.6 and 1.0% in infant formula (Figure 2C). These other lipids are of high interest as potential bioactive lipids with highly important functional roles in early life, many of which are clearly enriched in human milk compared to infant formula. The substantial differences in early life dietary lipids may contribute to why breastfed infants have increased risk protections when compared to formula-fed infants (6, 28).

Ether lipids are one of the ‘other’ lipid classes found in much higher (typically more than 10-fold) abundance in human milk than infant formula (Figure 3B). Ether lipids in human milk were first identified in human milk in the 1970s, and have been the subject of increasing interest in early life research in recent years (13, 28, 30–32). In the limited studies that have previously covered ether lipids, species are commonly presented as peak area and/or relative to other species, not quantified. Previous work in BIS has shown that breastfeeding is positively associated with 90% of infant circulating lipids at 6 months of age, including ether lipids, some of which were up to 19-fold higher in breastfed infants than formula-fed infants at 6 months of age (18). Alkyldiacylglycerols (TG(O)) are the major ether lipid class (Figures 3A,C), which make up approximately 75% of the ether lipid composition and approximately 0.4% of the total human milk lipidome, but only <0.1% of the total lipids in infant formula (Figure 2C). To accurately quantitate the AKG composition of TG(O), samples were saponified (Figure 4), and AKG(18:0), AKG(16:0), and AKG(18:1) containing TG(O) were the most abundant, these three AKG species have previously been identified in similar concentrations in a single human milk sample (12), among many other long chain saturated and monounsaturated species. In mice, milk ether lipids are broken down into AKG and metabolised to platelet-activating factor by adipose macrophages, activating the IL-6/STAT3 signaling pathway, and impeding the conversion of beige adipose into white adipose tissue in the pups. Shortened presence of beige adipose tissue results in increased white adipose tissue accumulation, leading to a higher risk of obesity later in life. Thus, formula-fed infants may be missing out on many essential AKG that protect against obesity development. Higher amounts of beige adipose tissue have also been measured in breastfed infants, suggesting that this mechanism through which AKG are sustaining beige adipose and impeding early accumulation of white adipose, is occurring in humans (12). While the presence of TG(O) in infant formula was somewhat surprising, as it has not been published before, concentrations were significantly (6–10 fold) lower than those in human milk. There were many individual TG(O) species that we found in human milk that were not present in infant formula at all (including TG(O-48:1) and TG(O-54:4)). Further, the most abundant resulting alkylglycerols, AKG(16:0), AKG(18:0), and AKG(18:1) were essentially negligible in formula in comparison (Figure 4C). We also found that, of the species that were present, they were much lower than in human milk (such as TG(O-54:2) which was up to 100-fold lower). Lactating rats supplemented with AKG resulted in milk with higher AKG than those that were not (33). Human supplementation studies have shown that supplementation with specific AKG has significant impact on circulating and cellular plasmalogens, hence it will be important to define the exact functions and roles of specific TG(O) species in order to translate this work into early life supplementation to allow optimal health benefits (34).

Dietary TG(O) are known precursors to plasmalogens. Plasmalogens, alkenyl phosphatidylethanolamines (PE(P)), are the second-most abundant ether lipid class in human milk (Figure 3D). These are highly bioactive lipid species, and their unique structure allows them roles as antioxidants, in cell differentiation, in lipid regulation, and in metabolism. Circulating plasmalogens are lowered in obesity, type 2 diabetes, and other disease states in humans (35–37). Indeed, evidence is emerging on the role of plasmalogens in early life - total PE(P) has been negatively associated with fat mass and positively associated with free-fat mass, and alkenyl phosphatidylcholine (PC(P−18:0/18:0)) has been linked to preterm infants growing on a fast trajectory (13, 27, 31). Plasmalogens are a reservoir of long chain PUFAs, which we found to be the case in human milk also, with species including PE(P−18:1/22:6), and PE(P−16:0/22:4) (32). It is of interest that plasmalogens are abundant in the adult brain, yet relatively low in the newborn brain (38). Studies have shown that formula-fed infants have poorer cognitive outcomes than breastfed infants (39, 40). In formula supplementation studies, addition of DHA and AA, which are typically esterified on TG, did not improve cognitive function to the level of breastfed infants (41). These results suggest that the lipid species that carry the PUFAs are critical to ensure they contribute to the appropriate signalling mechanisms, propelling the need to understand the role of plasmalogens in early life and those present in human milk and breastfed infants.

While ether lipids were higher in human milk than in infant formula, the high variation exhibited between individuals was notable, and thus intake was also highly variable between exclusively breastfed infants (Figure 6). The variability of the human milk lipidome has been shown many times, with the lipid profile likely a combination of diet and genetics, as well as the total fat content of the sample (Supplementary Tables S9–S12) (2). While milk synthesis remains somewhat a mystery, lactating cells have been shown to express relevant genes involved in vinyl-ether addition (PEDS1) and fatty acid to fatty alcohol conversion (FAR1 and FAR2), and thus potentially have notable ether lipid synthesis capability (42, 43). It is unlikely that maternal diet contains ether lipids in appreciable amounts, but maternal diet will provide precursors such as PUFAs. Differential synthesis and levels in human milk may also contribute to the unclear relationship between breastfeeding and disease risk, as levels may not be fed to all breastfed infants in sufficient amounts.

### Human milk lipids exhibit longitudinal trends

Longitudinal changes in the human milk lipidome are thought to occur to suit the infants’ changing needs and have potential biological relevance, as well as implications for sampling in birth cohorts. This is clearly evidenced by the vast concentration increases between pre- and post-feed samples, such as TG species increasing up to 12-fold, adding complication to human milk studies. We identified several differences through lactation, from birth to 6 months, in the UWAC. Colostrum is critical for immune protection and development, and often considered low in lipids and energy, with smaller milk fat globules and high in immune factors and hormones (44). Our findings indicate that in fact, the total lipid content is the same in both colostrum and mature milk, and many potentially bioactive lipids are very high in concentration in colostrum, compared to mature milk at 1 month. Previously, TG(O) have also been identified to be significantly higher in colostrum (28). Though the infant receives a very small volume of colostrum in the first hours to days of life, the highly bioactive functions of these lipids may be critical. Ether lipids have been linked to immunity in adults, having structural and functional importance in immune cells, signposting the possibility of their role in early life immune protection (35, 45, 46).

Significant differences in both lipid concentrations and relative abundance were identified from months one to six of lactation. Indeed, longitudinal changes in human milk composition have been previously identified in bioactive components, including in human milk oligosaccharides (47). Relative abundance of TG(O), for example, did not change throughout lactation (1 to 6 months), while total PE(P) decreased. PE(P) species as a total of PE have been previously shown to decrease (13). While there is little data on lipid digestion, absorption, and metabolism in early life, it is possible that this is due to changes in infant requirement with an evolving gastrointestinal system – as pH decreases, enzymatic activity increases, and the intestinal barrier develops (48). AKG resulting from TG(O) lipolysis would survive even the low pH (as they do in adult supplementation), while PE(P) would be destroyed. In contrast, infant formula composition will not change over lactation. Compositional changes are not only relevant in understanding differences between breastfed and formula-fed infants. Some infants, including preterm infants or those who are very ill, receive donor milk. In Australia, lactating volunteers provide milk which is pooled and provided to these vulnerable infants. Depending on the time of donation, milk may not contain the required bioactive lipids for that infant. The exact biological relevance of this is yet to be understood, however, because these infants are more vulnerable than their term counterparts, it is an essential consideration.

### The human milk lipidome impacts infant circulating lipids

Positive correlations were identified between matched human milk and infant plasma lipids, with ether lipids accounting for 40% of the significantly correlated lipids. Previously, some human milk TG, PE, and FFA species have been correlated with infant circulating PC(O-36:4) (49). Nutrient transfer from mother to infant has been of interest for many other species and ratios (such as DHA and LA), and this novel finding suggests that dietary lipids may impact development of infant circulating ether lipids, which is essential to understand because we know that metabolic physiology is established early in life (7, 50). These results have important implications for infant nutrition and health, as they suggest that increasing the levels of ether lipids in maternal diet could lead to increased human milk ether lipid content and potentially alter infant circulating ether lipids. This could have downstream effects on infant metabolic health and disease risk, as early-life lipid metabolism has been linked to the development of chronic diseases such as obesity and type 2 diabetes later in life (51–53).

### Strengths and limitations

To date, this is the most comprehensive human milk lipidomics study, utilising advanced lipidomics methodology to interrogate the complexity of the human milk lipidome. This study included a large sample size of human milk samples (*n* = 654), from both exclusively breastfeeding and mixed feeding dyads, allowing us to capture the variability of the lipidome, and compare it with infant formula and animal milk to explore differences that may contribute to infant health. The study’s focus on ether lipids is novel and not able to be conducted in many biological samples due to their low abundance. Furthermore, this is the first example of extensive dietary lipid intake differences in early life, between breastfed and formula-fed infants, and is a novel way to consider potential subsequent health differences.

This research was limited in that health outcomes were not analysed, which is a key next step. Other milk components were also not considered, which may also have health implications. Consideration of a combination of different milk components will be a critical step to fully comprehend human milk composition and the role of human milk and breastfeeding in early life. Although our findings were comparable to the limited work published already, it is important to consider that (1) samples were stored for 24 h (fridge or freezer) prior to being stored at −80°C, and that for some participants this was done in their own home thus samples may have been subject to conditions that influenced lipid composition, (2) that timing between milk samples and infant plasma samples were not optimally timed and infant intake was not able to be considered, and thus correlation analyses were simple and limited, and (3) all study participants were based in Australia, thus potential ethnic, or more likely dietary, differences may mean that these findings are not representative of the entire world.

## Conclusion

While many bioactive lipids found in human milk have been identified, there is still much to learn about their specific functions and how they contribute to the differences observed in health outcomes between breastfed and formula-fed infants. Ether lipids, which are present in higher concentrations in human milk compared to formula, may play an important role in infant health. Given the significant differences in the lipidome between human milk and formula, it is not surprising that formula-fed infants do not receive the same protections as breastfed infants. To address this issue, further research is needed to understand the specific role of human milk bioactive lipids in early life, for it to be translated into maternal supplementation, donor milk supplementation, or improvements in infant formula composition. Continuation of this research is essential to ensure that all infants have the best possible start in life.

## Data availability statement

The original contributions presented in the study are included in the article/Supplementary material, further inquiries can be directed to the corresponding author.

## Ethics statement

This study received ethical approval from the Barwon Health Human Research and Ethics Committee (HEC 10/24). Ethics approval was obtained by The UWA Human Research Ethics Office (RA/4/20/4023), and all participants provided informed written consent. The studies were conducted in accordance with the local legislation and institutional requirements. Written informed consent for participation in this study was provided by the participants’ legal guardians/next of kin.

## Author contributions

AG and DG: UWAC project administration. RS, PV, A-LP, and DB: BIS project administration. AG, SP, TW, NM, TD, KH, CG, AN, SB, and PM: methodology. AG and SP: acquisition and analysis. AG, TW, SB, and PM: interpretation. AG, TW, and SB: manuscript writing. AG, SP, TW, NM, TD, KH, CG, AN, DG, TM, RS, PV, A-LP, DB, SB, and PM: manuscript revision. AG, SB, and PM: funding acquisition. All authors contributed to the article and approved the submitted version.

## Group member of Barwon Infant Study Investigator

Anne-Louise Ponsonby, David Burgner, Fiona Collier, John Carlin, Katie Allen, Mimi Tang, Peter Sly, Peter Vuillermin, Richard Saffery, Sarath Ranganathan, Terry Dwyer.

## Funding

AG is supported by International Society for Research in Human Milk and Lactation—Family Larsson-Rosenquist Foundation funding. KH is supported by a National Health and Medical Research Council (NHMRC) investigator grant (1197190). DG receives an unrestricted research grant from Medela AG, administered by the University of Western Australia. TM is supported by an early-career fellowship from the Murdoch Children’s Research Institute. PV is supported by an NHMRC Career Development Fellowship. A-LP is supported by an NHMRC Investigator Grant. DB is supported by an NHMRC Investigator Grant. PM is supported by an NHMRC Investigator grant (2009965). This work was supported by LEW Carty grant and by the Victorian Government’s Operational Infrastructure Support Program. The funding bodies had no input in design or publication of this study.

## Conflict of interest

DG declares a potential conflict of interest (DG receives an unrestricted research grant from Medela AG, administered by the University of Western Australia). PM declares a potential conflict of interest (PM has licenced plasmalogen precursor supplement IP to Juvenescence Ltd.).

The remaining authors declare that the research was conducted in the absence of any commercial or financial relationships that could be construed as a potential conflict of interest.

## Publisher’s note

All claims expressed in this article are solely those of the authors and do not necessarily represent those of their affiliated organizations, or those of the publisher, the editors and the reviewers. Any product that may be evaluated in this article, or claim that may be made by its manufacturer, is not guaranteed or endorsed by the publisher.
