# Supplementary material for: Defining the lipid profiles of human milk, infant formula, and animal milk: implications for infant feeding
Source: Front Nutr. 2023 Aug 30;10:1227340. doi: 10.3389/fnut.2023.1227340 (PMC10499237; doi:10.3389/fnut.2023.1227340)
Supplement: Supplementary file 1 [file Data_Sheet_1.PDF]

Defining the lipid profiles of human milk, infant formula, and animal milk: implications for infant feeding

Supplementary Files

Supplementary File S1 – Butanol:methanol extraction comparison with chloroform:methanol

Method: Human milk replicate (n=4) samples (10 µL) were extracted in triplicate with butanol:methanol (1:1, v/v) and with chloroform:methanol (2:1) for comparison (1,2). Human milk lipids were extracted from 10 µL human milk samples using 100 µL extraction solvent containing 10 mM ammonium formate and internal standards. Samples were vortexed, sonicated for 1 hour, centrifuged for 10 minutes (14000x *g*, 20°C), and supernatant was transferred to 2 mL glass mass spectrometry vials with 250 µL inserts for analysis (Agilent Technologies). These extracts were analysed on an Agilent 1290 ultra-high performance liquid chromatography (UHPLC) system coupled to an Agilent 6490 QQQ mass spectrometer, using our established plasma lipidomics method (1). The high levels of triacylglycerols (TG) in human milk were supressing the TG signal, therefore samples were diluted for separate TG analysis, to ensure accurate quantification. All samples were extracted using butanol:methanol, with some alterations (described for each analysis method).

Results: In general, butanol:methanol extraction matched lipid extraction with single-phase chloroform:methanol. The mean±standard deviation (1.04±0.32-fold) and median (0.96-fold) differences between butanol:methanol and chloroform:methanol were not significantly different (Supplementary Table 1). Replicate coefficient of variation (CV) was higher ($p<0.0001$) for chloroform:methanol (mean 28%; median 17%) than butanol:methanol (mean 18%; median 15%). TG measurement of undiluted samples exhibited high variation, due to detector saturation, thus for TG analysis samples were diluted (1 in 100) with milliQ water and analysed in a separate method. The recovery of all lipid standards spiked in human milk using butanol:methanol was >90%.

Supplementary File S2 - Lipidome LC-MS analysis

Lipidome analysis was carried out on an Agilent 1290 UHPLC system, with a single column, set to 45°C (ZORBAX eclipse plus C18 column: 2.1 x 100 mm, 1.8 µm, Agilent), coupled with an Agilent 6495C triple quadrupole mass spectrometer. Samples were extracted with butanol:methanol, as in *Lipid extraction.* Lipid extracts were placed in autosampler tray (20°C) and 1 µL was injected. Lipids were separated with a stepped linear gradient of solvent A (50% water / 30% acetonitrile / 20% isopropanol, v/v/v) and solvent B (1% water / 9% acetonitrile / 90% isopropanol, v/v/v), both containing 10 mM ammonium formate, at a flow rate of 0.4 mL/min. The solvent gradient started at 85% A, decreasing to 50% over 2.5 min, to 43% over 0.1 min, to 30% over 6.4 min, to 7% over 0.1 min, to 4% over 1.9 min, then to 0% over 0.1 min and held for 0.9 min, then increased back to 85% over 0.2 min and held for 3.8 min to equilibrate the column prior to the next injection. The following mass spectrometer conditions were used; gas temperature, 150°C, gas flow rate 17 L/min, nebulizer 20 psi, sheath gas temperature 200°C, capillary voltage 3500 V and sheath gas flow 10 L/min. Concentrations for each lipid species were calculated based on area under the chromatographic curve relative to the labeled internal standard concentrations (1). For the UWAC samples, the chromatography was extended over 20 minutes (gradient: start at 85% solvent A, decrease to 50% over 2.5 min, decrease to 43% over 0.1 min, decrease to 30% over 6.4 min, decrease to 15% over 0.1 min, decrease to 0% over 6.9 min, hold at 0% for 1 min, decrease back to 85% over 0.1 min and hold to equilibrate for 2.9 min), and retention time windows shifted appropriately, to include lower-abundance short chain fatty acid containing TG (SCFA-TG) with the lipidome.

Internal standards used for the lipidome method: AC 16:0 d3, CE 18:0-d6, Cer(d18:1-d7/18:0), COH-d7, Cholic Acid d4, DG(15:0 18:1) d7, dhCer 8:0, FA(18:1) d9, Hex2Cer(d18:1/15:0) d7,

Defining the lipid profiles of human milk, infant formula, and animal milk: implications for infant feeding

Supplementary Files

HexCer(d18:1/15:0) d7, LPC(18:1) d7, LPE(18:1) d7, LPI 13:0, MG(18:1) d7, PA(15:0_18:1) d7, PC(15:0_18:1) d7, PC(P-18:0/18:1) d9, PE(15:0_18:1) d7, PE(P-18:0/18:1) d9, PG(15:0_18:1) d7, PI(15:0_18:1) d7, PS(15:0_18:1) d7, S1P(d18:1) d7, SM(d18:1/15:0) d9, Sph(d17:1), SHexCer(d18:1/12:0), TG(48:1) d7, Hex3Cer(d18:1/17:0), BMP(14:0/14:0), CL(56:0).

Supplementary File S3 - Triacylglycerol LC-MS analysis

Because the concentration of triacylglycerols in milk were so high relative to other lipid species, a separate analysis was performed where samples were diluted (1 in 100) with milliQ water before lipids were extracted from 10 µL with butanol:methanol, as described above. Analysis of milk triacylglycerols was performed on an Agilent 6490 QQQ mass spectrometer with an Agilent 1290 series UHPLC system and a ZORBAX eclipse plus C18 column (2.1 x 100 mm, 1.8 µm, Agilent) set at 45°C. Mass spectrometry analysis was performed in positive ion mode. The solvent system consisted of solvent A: 50% H_2O / 30% acetonitrile / 20% isopropanol (v/v/v) containing 10 mM ammonium formate and solvent B: 1% H_2O / 9% acetonitrile / 90% isopropanol (v/v/v) containing 10 mM ammonium formate. The gradient was as follows; starting with a flow rate of 0.4 mL/minute at 75% B and increasing to 90% B over 10 minutes, and finally to 100% B over 0.5 minutes. The solvent was then held at 100% B for 2 minutes. Equilibration was as follows, solvent was decreased from 100% B to 75% B over 0.5 minutes and held for an additional 2 minutes. Total run time was 15 min. The following mass spectrometer conditions were used; gas temperature, 150°C, gas flow rate 17 L/min, nebulizer 20 psi, sheath gas temperature 200°C, capillary voltage 3500 V, and sheath gas flow 10 L/min. Concentrations of each triacylglycerol were calculated based on chromatographic peak area relative to the labelled triacylglycerol (TG(48:1) d7) internal standard (1).

Supplementary File S4 - Total fatty acids LC-MS analysis

For the analysis of total fatty acids, milk samples were saponified to release all fatty acids prior to analysis. Lipids were first extracted from 10 µL of a 1 in 10 dilution of the milk samples (diluted with milliQ water) using butanol:methanol (as above). The lipid extract (50 µL) was transferred to a glass tube and dried down under a stream of nitrogen. Then, 50 µL of tetrahydrofuran:methanol (1:1, v/v) was added to the dried extract, vortexed briefly and 400 µL of 1 M potassium hydroxide in methanol, the protic solvent, was added and incubated at 80°C for 2 hrs. Following this alkaline hydrolysis, tubes were left at room temperature for 5 min, and then 400 µL of 3 M citric acid, were added to the hydrolysate and incubated at room temperature for 5 min, to prevent methyl esterification (3). After this, 4000 µL of hexane was added, and the mixture was shaken in a rotary mixer for 10 min. MilliQ water (2000 µL) was then added and the mixture shaken for 10 min. The mixture was centrifuged at 14,000 x *g* for 5 min. The organic (upper) phase was then transferred to a new glass tube and dried down under a stream of N_2. H_2O saturated butanol (50 µL) and 50 µL of methanol containing 10 mM ammonium formate was added, briefly vortexed, sonicated for 10 min, centrifuged at 14,000 x *g* for 2 min and finally transferred to 200 µL micro-inserts in 32 x 11.6 mm glass vials with Teflon insert caps. Analysis was carried out on an Agilent 1290 UHPLC system, with a single column, set to 45°C (ZORBAX eclipse plus C18 column: 2.1 x 100 mm, 1.8 µm, Agilent), coupled with an Agilent 6495C triple quadrupole mass spectrometer. Saponified extracts were placed in autosampler tray (20°C) and 1 µL was injected. Lipids were separated with a stepped linear gradient of solvent A (50% water / 30% acetonitrile / 20% isopropanol, v/v/v) and solvent B (1% water / 9% acetonitrile / 90% isopropanol,

Defining the lipid profiles of human milk, infant formula, and animal milk: implications for infant feeding

Supplementary Files

v/v/v), both containing 10 mM ammonium formate, at a flow rate of 0.4 mL/min. The solvent gradient started at 85% A, decreasing to 50% over 2.5 min, to 43% over 0.1 min, to 30% over 6.4 min, to 7% over 0.1 min, to 4% over 1.9 min, then to 0% over 0.1 min and held for 0.9 min, then increased back to 85% over 0.2 min and held for 3.8 min to equilibrate the column prior to the next injection. The following mass spectrometer conditions were used; gas temperature, 150°C, gas flow rate 17 L/min, nebulizer 20 psi, sheath gas temperature 200°C, capillary voltage 3500 V and sheath gas flow 10 L/min. Concentrations of each fatty acid was calculated based on chromatographic peak area relative to deuterated fatty acid internal standard (FA(16:0)-d3, FA(18:1)-d9, FA(20:4)-d11, and FA(22:6)-d5) concentrations. The efficiency of saponification was assessed by the residual triacylglycerol in saponified samples.

Supplementary File S5 – Alkylglycerol LC-MS analysis

Because we noted that the amount of TG(O) species was significant in human milk, we also analysed the alkylglycerol composition to quantitate the total TG(O) species. Lipids were extracted from milk with butanol:methanol, as described. Following this, a portion of the lipid extract (80 µL) was dried under a constant stream of nitrogen. Then, 100 µL of 0.1 M sodium hydroxide in methanol, the protic solvent, was added to the dried extract and alkaline hydrolysis was carried out for 2 hours at 80°C. Following saponification, 10 µL of 1 M formic acid was added to stop the hydrolysis reaction. The hydrolysate was then dried under a constant stream of nitrogen and finally reconstituted with 200 µL butanol and methanol (1:1, v/v) (with 10 mM ammonium formate) containing a mixture of the internal standards. The extracts were mixed and stored at -80°C until further analysis. Analysis was carried out on an Agilent 1290 UHPLC system, with a single column, set to 45°C (ZORBAX eclipse plus C18 column: 2.1 x 100 mm, 1.8 µm, Agilent), coupled with an Agilent 6495C triple quadrupole mass spectrometer. Extracts were placed in autosampler tray (20°C) and 1 µL was injected. Lipids were separated with a stepped linear gradient of solvent A (50% water / 30% acetonitrile / 20% isopropanol, v/v/v) and solvent B (1% water / 9% acetonitrile / 90% isopropanol, v/v/v), both containing 10 mM ammonium formate, at a flow rate of 0.4 mL/min. The solvent gradient started at 85% A, decreasing to 50% over 2.5 min, to 43% over 0.1 min, to 30% over 6.4 min, to 7% over 0.1 min, to 4% over 1.9 min, then to 0% over 0.1 min and held for 0.9 min, then increased back to 85% over 0.2 min and held for 3.8 min to equilibrate the column prior to the next injection. The following mass spectrometer conditions were used; gas temperature, 150°C, gas flow rate 17 L/min, nebulizer 20 psi, sheath gas temperature 200°C, capillary voltage 3500 V and sheath gas flow 10 L/min. For quantification of alkyl glycerol species, a deuterated monoacylglycerol (MG 18:1d7) was used as an internal standard. Response factors for alkylglycerol species against MG 18:1d7 were calculated using serially diluted synthetic alkyl glycerol species in a range 1-300 µM and a fixed amount of MG 18:1 d7. The efficiency of saponification was assessed by the residual triacylglycerol in saponified samples.

References

1. Huynh K, Barlow CK, Jayawardana KS, Weir JM, Mellett NA, Cinel M, et al. High-Throughput Plasma Lipidomics: Detailed Mapping of the Associations with Cardiometabolic Risk Factors. Cell Chemical Biology. 2019;26(1):71-84.e4.
2. Weir, J. M., et al. Plasma lipid profiling in a large population-based cohort. Journal of Lipid Research. 2013;54(10): 2898-2908.

Defining the lipid profiles of human milk, infant formula, and animal milk: implications for infant feeding

Supplementary Files

3. Beal, G.D. Myristic Acid [Internet]. Place unknown: Organic Syntheses, Inc; Publication date unknown [updated 2023]. Available from: http://www.orgsyn.org/demo.aspx?prep=CV1P0379
